# Glomerular Injury Findings in Patients with Thalassemia Minor

**DOI:** 10.3390/ijms27073209

**Published:** 2026-04-01

**Authors:** Zufit Hexner-Erlichman, Erez Shamir, Basem Hijazi, Hanna Rosenbaum, Nayaf Habashi

**Affiliations:** 1 Central Pediatrics Clinic, Clalit Health Services Community Division, North District, Migdal HaEmek 2353021, Israel; 2 The Azrieli Faculty of Medicine, Bar-Ilan University, Safed 5290002, Israel; erez5928@gmail.com (E.S.); roseerlich@gmail.com (H.R.); habashinayaf@gmail.com (N.H.); 3 Hemato-Oncology and Gaucher Center, Nazareth Towers Center for Consultant Medicine, Clalit Health Services Community Division, North District, Nazareth 1621601, Israel; 4 Nephrology Center, Nazareth Towers Center for Consultant Medicine, Clalit Health Services Community Division, North District, Nazareth 1621601, Israel; 5 Ruth and Bruce Rappaport Faculty of Medicine, Technion—Israel Institute of Technology, Haifa 3200003, Israel

**Keywords:** β-thalassemia minor, renal involvement, hematuria, proteinuria, retrospective cohort, real-world analysis

## Abstract

Renal involvement in β-thalassemia minor (β-TMin) has been described mainly in case reports and small observational studies, and its clinical significance remains incompletely characterized. Using real-world data from routinely collected electronic medical records, we performed a retrospective cohort study including 1516 adult patients with β-TMin insured by Clalit Healthcare Services to explore renal abnormalities identified during routine clinical care. Urine testing for hematuria, microalbuminuria, and proteinuria was not performed systematically but was ordered at clinicians’ discretion, resulting in evaluation of a clinically selected subset of patients. Among those tested, hematuria, microalbuminuria, and proteinuria were commonly documented, often in the absence of hypertension, diabetes mellitus, congestive heart failure, or impaired kidney function, consistent with largely subclinical renal involvement. Patients who underwent urine testing were older and had more comorbidities than untested patients, indicating potential selection bias. Correlation analyses showed weak associations between hematological and renal parameters, while ferritin levels correlated modestly with selected proteinuria measures. Due to the retrospective design and non-systematic urine assessment, population-level prevalence and clinical impact cannot be determined. Therefore, prospective studies with standardized renal evaluations are needed to better characterize the frequency, mechanisms, and clinical relevance of renal abnormalities in β-TMin.

## 1. Introduction

Hemoglobinopathies are the most prevalent monogenic disorders globally, with 1–5% of the world’s population carrying a genetic mutation for thalassemia [1]. The exceptionally high prevalence of hemoglobin disorders compared to other monogenic disorders is influenced by natural selection, driven by the high rate of consanguinity in many countries and increased resistance of carriers to Plasmodium falciparum malaria [2]. Gene drift and founder effects are other reasons that thalassemias are most frequent in southeastern and southern Asia, the Middle East, the Mediterranean countries, and North and Central Africa [3,4]. However, increasing migration has led to a rising incidence worldwide, making their management and care an increasing concern for health care systems [3,4]. In Israel, β-thalassemia is panethnic and endemic, with a carrier rate of approximately 2.4%; the incidence is higher in primarily Arab towns [5].

Beta-thalassemias are a group of hereditary blood disorders characterized by anomalies in the synthesis of the beta chains of hemoglobin, resulting in variable phenotypes ranging from severe anemia to clinically asymptomatic individuals [6]. β-thalassemia is a monogenic disorder caused by a spectrum of mutations in β-globin gene (HBB), resulting in quantitative reduction in β-globin and accumulation of α-globin chains that are structurally normal. This results in globin chain imbalance, which is the basis of the pathophysiology of these disorders. The imbalance in the α/β-globin chain ratio leads to ineffective erythropoiesis, chronic hemolytic anemia of varying severity with compensatory hemopoietic expansion [7,8,9,10].

β-thalassemia includes three main forms: β-thalassemia major, also termed “Cooley’s anemia” or “Mediterranean anemia”. β-thalassemia major presents in early childhood with profound anemia necessitating blood transfusions for survival. Whereas β-thalassemia intermedia and β-thalassemia minor (β-TMin) are transfusion independent. β-TMin also termed “thalassemia trait”, is a symptomless carrier state of β-thalassemia. It may be diagnosed by the presence of microcytic hypochromic erythrocytes with mild anemia. The microcytosis is much more profound in β-TMin than in iron deficiency anemia [10,11].

The study of renal manifestations in patients with β-thalassemia has primarily focused on the potential nephrotoxic effects of iron chelators used to manage transfusional iron overload in β-thalassemia major patients; however, the direct impact of thalassemia on kidney function, particularly in β-TMin patients, has not been extensively evaluated. Renal tubular abnormalities are well described in β-thalassemia major and intermedia [12,13]. Several studies in children with β-TMin have reported renal tubulopathy [14,15]. Additionally, case reports and small cohort studies in adults with β-TMin have identified tubular dysfunction, characterized by hypercalciuria, renal magnesium and uric acid wasting, glucosuria, reduced phosphorus reabsorption, and tubular proteinuria [16,17,18,19,20]. Research on estimated glomerular filtration rate (eGFR) in thalassemia patients has yielded conflicting results. Some studies have reported abnormally elevated eGFR in individuals with β-thalassemia intermedia, with a declining trend observed as age advances [21]. In contrast, other studies have reported either a decrease or no significant change in eGFR among adult patients with β-thalassemia intermedia and major [22,23]. These variations have been attributed to factors such as chronic anemia, renal hemosiderosis, and the effects of iron chelation therapy [22,23].

By retrospectively reviewing medical records, this study aimed to determine the presence and prevalence of renal complications, including both tubular and glomerular dysfunction, in a large cohort of adult β-TMin patients insured by Clalit Healthcare Services, Israel’s largest HMO.

## 2. Results

A total of 1516 adult patients (1080 females and 436 males) were included in this study based on retrospective review of medical records. Baseline demographic, clinical, and hematological characteristics are summarized in Table 1 and Table 2. Patients ranged in age from 19 to 103 years (mean age at diagnosis: 44.65 ± 16.73 years), and the average age at the time of data collection was 40.34 ± 14.90 years. The mean hemoglobin level was 11.56 ± 1.79 g/dL, the mean corpuscular volume (MCV) was 71.43 ± 8.61 fL, the mean hemoglobin A_2_ was 4.82 ± 2.39%, the mean HbF level was 0.78 ± 1.15% and the mean ferritin level was 78.38 ± 66.37 ng/mL. The mean creatinine level was 0.70 ± 0.29 mg/dL. The mean estimated glomerular filtration rate (eGFR) was 104.49 ± 26.26 mL/min/1.73 m^2^.

Urine testing for hematuria, proteinuria, or microalbuminuria was performed in a clinically selected subset of patients and was ordered due to clinical indications and physicians’ judgment rather than as part of a standardized screening approach. Consequently, patients who underwent urine testing were significantly older, more frequently female, had lower hemoglobin levels, and had a higher prevalence of hypertension, diabetes mellitus, and congestive heart failure compared with untested patients (Table 3). Across individual urine assessments, tested patients consistently differed from those not tested—most notably by older age and a higher comorbidity burden—reflecting selective evaluation based on clinical judgment (Table S1).

**Figure 1:**
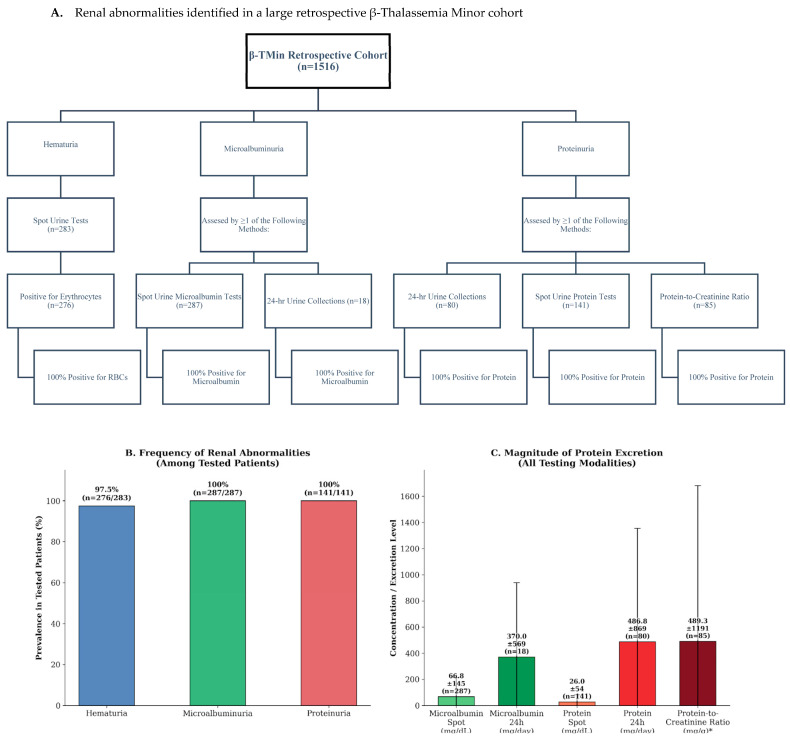
Distribution of renal abnormalities among patients who underwent urine testing. (**A**) Diagnostic algorithm and study flowchart showing the patient selection process. Note that urine testing was performed based on clinical indication. (**B**) Frequency of renal abnormalities among the subset of patients tested. Values indicate the percentage of positive findings for each parameter. (**C**) Magnitude of protein excretion across different testing modalities. The asterisk is a modality aimed to assess proteinuria. Data are presented as Mean ± Standard Deviation (SD), highlighting the variability in excretion levels. Abbreviations: RBCs, Red Blood Cells; SD, Standard Deviation.

Hematuria was assessed in 283 of the 1516 patients, based on retrospective review of their medical records, and was identified in 276 patients (97.5%). The majority of those with hematuria were female (222 patients, 80.43%), while 54 (19.56%) were male, yielding a male-to-female ratio of 0.24:1. Among the female patients with hematuria, 24% (53 individuals) were over the age of 50 and considered postmenopausal as presented in Figure 1A,B. Notably, 255 patients had no documented comorbidities such as hypertension, congestive heart failure, or diabetes mellitus, and retrospective chart data indicated that 97.3% of them exhibited hematuria.

Microalbumin measurements were identified through retrospective review of patients’ medical records. Spot urine microalbumin results were available for 287 patients, with a mean value of 66.79 ± 145.42 mg/dL (range: 3.7–1211.75 mg/dL). Among these, 18 patients also had documented 24-h urine collections for microalbumin assessment, showing a mean level of 370.04 ± 569.00 mg/dL (range: 3.6–1965.40 mg/dL). Microalbuminuria was identified in all patients (100%) from whom measurements were available, as shown in Figure 1. Based on retrospective chart data, 261 patients (90.9%) with positive spot urine microalbumin tests had no prior history of hypertension, and 257 (89.5%) had no history of diabetes mellitus. Similarly, among patients with positive 24-h urine results, 15 (83.3%) had no documented history of hypertension or diabetes mellitus.

Proteinuria-related data were obtained retrospectively from patients’ medical records across various testing modalities (see Figure 1). Documentation of 24-h urine collections was available for 80 patients, all of whom (100%) had proteinuria, with a mean protein level of 486.81 ± 868.98 mg/dL (range: 35.7–4657.30 mg/dL). Review of these patients’ medical charts showed that 76 (95%) had no history of hypertension, 74 (95.2%) had no diabetes mellitus, and 78 (97.5%) had no congestive heart failure. Spot urine protein measurements were available for 141 patients, all of whom (100%) demonstrated proteinuria, with a mean level of 25.99 ± 54.27 mg/dL (range: 2.33–394.56 mg/dL). Among these patients, retrospective data indicated that 130 (92.2%) had no history of hypertension, 126 (89.4%) had no diabetes mellitus, and 134 (95%) had no congestive heart failure. Protein-to-creatinine ratio results were retrospectively recorded for 85 patients, all of whom (100%) exhibited proteinuria, with a mean protein level of 489.27 ± 1191.21 mg/dL (range: 40.69–8461.12 mg/dL). Among this group, 75 patients (88.2%) had no documented hypertension, 73 (85.9%) had no documented diabetes mellitus, and 79 (92.9%) had no documented congestive heart failure in their medical charts.

Review of patients’ medical records showed that none had undergone kidney biopsy.

Hematuria and microalbuminuria were concurrently identified through retrospective review of the medical records of 84 patients. Among these, 27 patients demonstrated both hematuria and proteinuria based on documented 24-h urine collections, while 51 patients showed hematuria and proteinuria in recorded spot urine tests. Additionally, 14 patients were noted to have hematuria and proteinuria based on a protein-to-creatinine ratio greater than 200.

**Figure 2:**
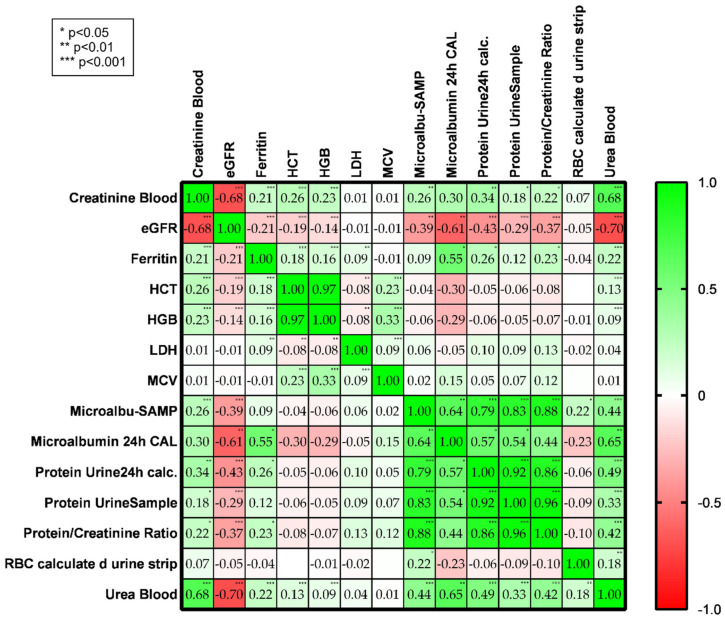
Correlation Analysis of hematological and renal parameters in adult β-Tmin patients. Pearson correlation matrix showing the relationships between hematological and renal parameters in adult patients with β-TMin. Each cell displays the Pearson correlation coefficient (r), with color coding indicating the strength and direction of the correlation: green for positive correlations and red for negative correlations. The color intensity reflects the magnitude of the correlation (scale bar on the right). Statistically significant correlations (*p* < 0.05) are marked with asterisks (*).

Weak, non-significant negative correlations were observed between hemoglobin, hematocrit, and MCV with eGFR, urine microalbumin, protein, and hematuria parameters (Figure 2). Ferritin levels demonstrated weak but statistically significant positive correlations with protein levels in 24-h urine collections (r(23) = 0.611, *p* = 0.002) and the protein/creatinine ratio (r(52) = 0.641, *p* < 0.001).

## 3. Discussion

Using real-world data from routinely collected electronic medical records, this study describes glomerular abnormalities observed in a large cohort of adult β-TMin patients who underwent urine testing in clinical practice. Renal abnormalities including hematuria, microalbuminuria, and proteinuria, were identified through retrospective review of records from a clinically selected subgroup, reflecting physician-directed evaluation and not protocolized screening. These findings indicate that renal abnormalities may be encountered in some adult β-TMin patients in real-world settings and warrant further investigation using systematic study designs.

Non–transfusion-dependent thalassemias are increasingly recognized as conditions associated with substantial long-term morbidity despite the absence of regular transfusion requirements. Chronic ineffective erythropoiesis and hemolysis contribute to iron dysregulation, hypercoagulability, and progressive multi-organ involvement, including vascular, hepatic, endocrine, and renal complications that may accumulate with age [24]. Within this spectrum, β-TMin is often perceived as a milder phenotype; however, these observations highlight that NTDT-related complications may extend, to a lesser extent, to β-TMin and may remain clinically silent without targeted evaluation.

The introduction of iron chelation therapy for patients with transfusion-dependent thalassemia over the past decades has prolonged survival and improved quality of life. However, with increased longevity, it has been accompanied by the emergence of previously underrecognized complications, including renal abnormalities. Reported renal manifestations in transfusion-dependent thalassemia include tubular and glomerular dysfunction, microscopic hematuria, Fanconi syndrome, and nephrolithiasis [25].

The precise mechanisms underlying renal dysfunction in patients with transfusion-dependent thalassemia are not yet fully understood. Several hypotheses have been proposed, including reduced systemic vascular resistance resulting from anemia and increased glomerular filtration as compensatory response. These alterations may adversely affect podocytes and mesangial cells, contributing to glomerulosclerosis and subsequent decline in GFR [25,26,27]. In patients with β-thalassemia major, renal tubular dysfunction is primarily attributed to iron overload from repeated blood transfusions and the nephrotoxic effects of iron chelation therapy [28,29]. Notably, none of the patients in the present cohort received blood transfusions during the study period, indicating that the observed renal abnormalities cannot be attributed to transfusion-related iron overload or chelation exposure.

Data on renal complications in thalassemia intermedia, transfusion-independent form of the disease, remain limited, and evidence regarding β-TMin is even more scarce. Three primary pathophysiological mechanisms have been proposed in thalassemia intermedia: ineffective erythropoiesis leading to increased intestinal iron absorption and iron overload, chronic anemia and tissue hypoxia, and both intra- and extravascular hemolysis [30,31]. We propose that these pathophysiological mechanisms are likely to be involved, to some extent, in β-TMin.

Evidence for renal tubular involvement in β-TMin derives primarily from small, single-center cohort studies. In limited pediatric and adult series, abnormalities such as proteinuria, microalbuminuria, glucosuria, and increased fractional excretion of uric acid and potassium have been reported in a subset of β-TMin patients, while eGFR remained preserved [32,33]. Although based on relatively small cohort sizes, these observations support the concept that renal involvement in β-TMin may be subclinical and underrecognized and support consideration of further renal evaluation.

In this study, the clinically selected subset of patients who underwent urinalysis, hematuria, indicated by the presence of red blood cells in the urine, was identified in the vast majority of tested patients based on retrospective evaluation of documented results. Most of these patients were females (approximately 80%), predominantly of reproductive age. This distribution likely reflects the routine preconception and prenatal carrier screening for Thalassemia in Israel, which commonly includes a complete blood count and may lead to earlier or more frequent recognition of β-TMin among women. The retrospective nature of the data suggests that this sex difference reflects differential screening exposure rather than increased disease burden. Notably, nearly one-quarter of women with hematuria were over the age of 50, indicating that hematuria was also observed among postmenopausal women, independent of reproductive-related screening practices and menstrual-cycle–related changes.

Urine microalbumin and protein measurements were available only for a subset of the cohort. Within this group, microalbuminuria was assessed using retrospectively reviewed spot urine tests and 24 h urine microalbumin collections, while proteinuria was evaluated through retrospective review of spot urine protein measurements, urine protein-to-creatinine ratios, and 24 h urine protein collections; microalbuminuria and proteinuria were identified in all tested patients. Importantly, retrospective review of the medical records showed that the majority of these patients had no documented comorbidities, including hypertension, diabetes mellitus, or congestive heart failure.

A relatively small proportion of patients with β-TMin in our cohort had documented urine testing for hematuria or proteinuria based on retrospective review of their medical records. Urinary evaluation was not performed systematically and was ordered at the discretion of treating physicians rather than through protocolized screening. As demonstrated by baseline comparisons (Table 2 and Table S1), patients who underwent urine testing were older and had a higher burden of comorbidities, suggesting preferential testing of individuals perceived to be at higher renal risk. Consequently, the observed frequencies of hematuria, proteinuria, and microalbuminuria reflect conditional prevalences within a clinically selected subgroup and should not be interpreted as estimates of prevalence in the overall β-TMin population. Importantly, the limited and indication-driven nature of testing suggests that the detected renal abnormalities may be attributable, at least in part, to underlying clinical conditions or risk factors that prompted testing, rather than to the β-thalassemia trait itself. Our findings are in line with recent ASH 2025 data suggesting that the risk of comorbidities in β-thalassemia carriers may be overestimated in hospital-based cohorts, likely due to selection bias, whereas studies using consecutively genotyped populations do not demonstrate an increased comorbidity risk [34]. Nevertheless, the consistent detection of renal abnormalities in this real-world setting suggests that renal involvement in β-TMin may be under-recognized and supports the need for prospective, systematic screening studies.

Levels of microalbumin, protein, and red blood cells (RBCs) in urine showed positive correlations with hemoglobin, hematocrit, and MCV (Figure 1). However, these associations were weak according to Pearson correlation analysis and did not reach statistical significance. This may reflect the limited number of proteinuria and hematuria assessments performed in β-TMin patients within our cohort. Notably, there have been studies reporting high sodium, potassium, magnesium, uric acid and phosphorus to creatinine ratios among β-thalassemia major and intermedia patients [35], and high urinary calcium excretion was found among β-TMin patients, some of them even showed an increased incidence of osteoporosis [36].

In the present study, ferritin levels showed statistically significant but weak positive correlations with both protein excretion in 24 h urine collections and the protein/creatinine ratio (Figure 1). Although modest in magnitude, these associations were consistent across different measures of proteinuria, suggesting a potential link between iron-related pathways and renal protein handling in adult patients with β-TMin. Ferritin may reflect iron metabolism dysregulation, chronic inflammation, or oxidative stress, all of which have been implicated in glomerular and tubular injury leading to increased urinary protein excretion [37]. Supporting this biological plausibility, prior studies have reported associations between ferritin levels and microalbuminuria in children and adolescents with type 1 diabetes mellitus [38] as well as in adults with type 2 diabetes mellitus [39]. Although causality cannot be inferred, these observations point to a possible involvement of iron-related pathways in renal abnormalities, warranting further investigation in patients with β-TMin. Collectively, these findings highlight the need for larger studies incorporating comprehensive renal phenotyping to better clarify the nature and clinical relevance of these associations.

A key limitation of our study is its retrospective, real-world design, which resulted in incomplete data capture and non-systematic renal assessment. Urine testing was performed in a clinically selected subset of patients and was guided by physician judgment rather than protocolized screening, limiting the ability to draw conclusions regarding the prevalence of renal abnormalities in the broader β-TMin population. The retrospective nature of the data precluded differentiation between transient and persistent urinary abnormalities, and residual confounding from unmeasured or incompletely captured variables cannot be excluded. In addition, no patients underwent kidney biopsy, reflecting both preserved kidney function and lack of clinical indications for invasive evaluation. Finally, reliance on electronic medical record-based diagnoses may not allow for complete distinction between β-TMin and thalassemia intermedia in all cases. Accordingly, larger prospective studies with standardized diagnostic criteria and systematic renal evaluation are required to better define the scope and clinical relevance of renal involvement in this population.

Future investigations should incorporate comprehensive and protocolized assessment of both tubular and glomerular function, including urinary electrolyte excretion, albuminuria and proteinuria indices, and appropriate imaging modalities such as renal ultrasonography, with advanced imaging or renal biopsy reserved for selected clinical indications. Such approaches would allow more accurate characterization of renal abnormalities in patients with β-TMin and help determine whether targeted renal monitoring should be considered in specific subgroups rather than assumed as a universal feature of the condition.

## 4. Materials and Methods

This retrospective, descriptive study was conducted between 2014 and 2023 and included 1516 adult patients with β-TMin insured by Clalit Healthcare Services. The diagnosis of β-TMin was based on documentation in the patients’ electronic medical records and supported by hemoglobin electrophoresis data extracted from these records. Genetic data were not available for this cohort, as genetic testing for β-thalassemia in Israel is not routinely performed and is primarily conducted within premarital screening programs involving couples in whom both partners have β-TMin. During the study period, none of the patients in the cohort received blood transfusions. All data were retrieved retrospectively and relied solely on existing electronic medical records. Extracted variables included demographic characteristics, detailed medical history with documentation of comorbidities such as hypertension, diabetes mellitus, and congestive heart failure (as defined by the International Classification of Diseases, Ninth Revision [ICD-9]), and diagnostic laboratory parameters (e.g., complete blood count and kidney function indices). All information was systematically recorded using a standardized data-collection tool. Data were reviewed for accuracy and completeness by trained personnel. To ensure confidentiality, patient identifiers were anonymized, and all procedures adhered to institutional ethical guidelines. The study protocol was approved by the local institutional review board of Clalit Healthcare Services (approval number 0165-23).

Glomerular filtration rate (GFR), calculated using the CKD-EPI equations, was retrieved from patients’ medical records.

Urine testing for hematuria, proteinuria, and microalbuminuria was not performed systematically across the cohort but was ordered at the discretion of treating physicians as part of routine clinical care. Consequently, only a subset of patients underwent urine evaluation, and comparisons between patients who underwent urine evaluation and those who did not were conducted to assess potential selection bias.

Proteinuria was assessed by collecting data on several parameters, including values of protein in urine spot exam, ratio of protein to creatinine also measured in urine spot testing and protein level in 24 h urine collection. Microalbuminuria was evaluated by gathering information on tests such as microalbumin levels in a urine sample and a 24 h urine collection. Hematuria was evaluated by checking for the presence of red blood cells in urinalysis tests.

Urine dipstick analyses were performed using automated urine chemistry analyzers (iChemVELOCITY or DxU Urine Chemistry Systems; Beckman Coulter, Brea, CA, USA) with iChemVELOCITY reagent strips. In cases of abnormal dipstick findings, urine microscopy could be performed at the discretion of the treating clinician and when documented in the medical record; however, urine microscopy data were not available for analysis in this cohort. Abnormal urinary findings were defined according to institutional reference ranges, including spot urine protein concentration > 15 mg/dL, protein-to-creatinine ratio > 200 mg/g, red blood cell count > 5 cells per high-power field, and 24 h urine protein excretion > 150 mg/24 h.

### Statistical Methods

The statistical analysis was performed using SPSS version 28 with a significance level set at 5% (*p* ≤ 0.05) and 80% statistical power. Categorical data were presented as frequencies and percentages (%) in frequency tables, while continuous data were assessed for normality using Shapiro–Wilk tests and Q-Q plots, with normally distributed variables reported as mean ± standard deviation (SD) and non-normally distributed variables reported as median with interquartile range (IQR). The dependence between two categorical variables was tested using the Chi-square test or Fisher’s exact test when expected cell counts were below 5. The relationship between continuous variables was examined using Pearson’s correlation coefficient for normally distributed data and Spearman’s rank correlation coefficient for non-normally distributed data. All statistical tests were two-tailed and interpreted with 95% confidence intervals, maintaining the predefined significance threshold and power requirements throughout the analysis.

## 5. Conclusions

This large retrospective, real-world cohort study suggests that renal abnormalities, including hematuria, microalbuminuria, and proteinuria, may be encountered in adult patients with β-TMin during routine clinical care. Importantly, these findings were largely identified in patients with preserved kidney function and in the absence of overt renal symptoms or common comorbidities, suggesting that renal involvement in β-TMin is often subclinical.

Previous case reports and small observational studies have documented renal findings in patients with β-TMin. The present analysis extends this body of evidence by evaluating such abnormalities within a substantially larger cohort, using routinely collected electronic medical records, thereby reinforcing the notion that β-TMin may not be a completely benign condition, particularly with respect to kidney involvement. However, given the retrospective design and the non-systematic, clinician-directed approach to urine testing, these findings do not allow estimation of population-level prevalence or firm conclusions regarding clinical significance and should be interpreted as conditional observations within a clinically selected subgroup.

Nonetheless, the recurrent detection of largely subclinical renal abnormalities in this real-world setting suggests that kidney involvement in β-TMin may remain underrecognized in routine practice. Further prospective studies with standardized and comprehensive renal assessments are needed to better characterize the frequency, mechanisms, and long-term clinical relevance of these findings, and to determine whether targeted renal evaluation may be warranted in selected patients.

## Figures and Tables

**Table 1:** Baseline demographic and clinical characteristics of the study cohort.

Parameter	Value
Age, years	36 [29, 48]
Males	436 (28.7%)
Creatinine level, mg/dL	0.65 [0.55, 0.78]
Estimated Glomerular Filtration Rate (eGFR)	116.3 [103.8, 126.3]
Hypertension	41 (2.69%)
Diabetes Melitus	40 (2.63%)
Congestive Heart Failure	13 (0.85%)

Continuous data are medians and [IQR].

**Table 2:** Hematological parameters of the study cohort.

Parameter	Value
Hemoglobin A_2_, %	4.82 ± 2.39
Hemoglobin F, %	0.78 ± 1.15
Hemoglobin level, g/dL	11.56 ± 1.79
Mean corpuscular volume (MCV), fL	71.43 ± 8.61
Ferritin, ng/mL	78.38 ± 66.37

**Table 3:** Comparison of tested vs. untested patients across urine assessments.

Urine Test	Tested (n)	Older Age	Female Predominance	Lower Hb	Comorbidities
Hematuria (RBC)	276	✔	✔	✔	✔
Microalbumin (spot)	286	✔	✔		✔
Microalbumin (24 h)	18	✔	—	✔	✔
Protein (spot)	141	✔	✔	✔	✔
Protein (24 h)	80	✔	✔	✔	—
Protein/Creatinine Ratio	28	✔		✔	✔

Summary of the differences between patients who underwent urine testing and those who did not, across individual urine assessments. Check marks (✔) indicate a statistically significant difference (*p* < 0.05) between tested and untested patients for the specified characteristic, while dashes (—) indicate no statistically significant difference. “Older age” refers to a significantly higher mean age in tested patients. “Female predominance” indicates a higher proportion of female patients among those tested. “Lower Hb” denotes a significantly lower mean hemoglobin concentration in tested patients compared with untested patients. Comorbidities indicate a higher prevalence of hypertension, diabetes mellitus, and/or congestive heart failure among tested patients, respectively. Detailed numerical data and statistical comparisons for each urine test are provided in Table S1.

## Data Availability

The original contributions presented in this study are included in the article and Supplementary Materials. Further inquiries can be directed to the corresponding author.

## Supplementary Materials

The following supporting information can be downloaded at: https://www.mdpi.com/article/10.3390/ijms27073209/s1.
