# Supplementary material for: Glomerular Injury Findings in Patients with Thalassemia Minor
Source: Int J Mol Sci. 2026 Apr 1;27(7):3209. doi: 10.3390/ijms27073209 (PMC13073669; doi:10.3390/ijms27073209)
Supplement: Supplementary file 1 [file ijms-27-03209-s001.zip › ijms-4102184-supplementary.pdf]

Table S1A—Hematuria assessment (RBC urine strip)

Variable	Not tested	Tested	p-value
Age (years)	n=1,240; 39.60 ± 14.35	n=276; 43.41 ± 16.80	<0.001
Hemoglobin (g/dL)	n=1,235; 11.83 ± 1.64	n=276; 11.34 ± 1.43	<0.001
Ferritin	n=785; 71.15 ± 109.52	n=221; 87.72 ± 129.00	0.057
Hypertension	29 (2.3%)	10 (3.6%)	0.223
CHF	6 (0.5%)	7 (2.5%)	<0.001
Diabetes mellitus	29 (2.3%)	8 (2.9%)	0.586
Male sex	381 (30.7%)	54 (19.6%)	<0.001
Female sex	859 (69.3%)	222 (80.4%)	<0.001

Table S1B—Microalbuminuria assessment (spot urine microalbumin)

Variable	Not tested	Tested	p-value
Age (years)	n=1,230; 36.95 ± 12.25	n=286; 54.69 ± 16.61	<0.001
Hemoglobin (g/dL)	n=1,225; 11.70 ± 1.61	n=286; 11.89 ± 1.61	0.075
Ferritin	n=781; 64.34 ± 98.53	n=225; 111.08 ± 151.76	<0.001
Hypertension	13 (1.1%)	26 (9.1%)	<0.001
CHF	1 (0.1%)	12 (4.2%)	<0.001
Diabetes mellitus	7 (0.6%)	30 (10.5%)	<0.001
Male sex	331 (26.9%)	104 (36.4%)	<0.001
Female sex	899 (73.1%)	182 (63.6%)	<0.001

Table S1C—Microalbuminuria assessment (24-hour urine collection)

Variable	Not tested	Tested	p-value
Age (years)	n=1,498; 40.18 ± 14.83	n=18; 49.78 ± 17.79	0.007
Hemoglobin (g/dL)	n=1,493; 11.74 ± 1.61	n=18; 11.44 ± 1.53	0.0433
Ferritin	n=990; 74.60 ± 114.44	n=16; 86.90 ± 102.21	0.669
Hypertension	36 (2.4%)	3 (16.7%)	<0.001
CHF	12 (0.8%)	1 (5.6%)	0.03
Diabetes mellitus	34 (2.3%)	3 (16.7%)	<0.001
Male sex	429 (28.6%)	6 (33.3%)	0.662
Female sex	1,069 (71.4%)	12 (66.7%)	0.662

Table S1D—Protein/creatinine ration

Variable	Not tested	Tested	p-value
Age (years)	n=1,488; 39.92 ± 14.61	n=28; 60.18 ± 16.70	<0.001
Hemoglobin (g/dL)	n=1,483; 11.75 ± 1.61	n=28; 10.99 ± 1.65	0.014
Ferritin	n=979; 74.20 ± 114.34	n=27; 96.41 ± 109.61	0.319
Hypertension	35 (2.4%)	4 (14.3%)	<0.001
CHF	10 (0.7%)	3 (10.7%)	<0.001
Diabetes mellitus	32 (2.2%)	5 (17.9%)	<0.001
Male sex	426 (28.6%)	9 (32.1%)	0.684
Female sex	1,062 (71.4%)	19 (67.9%)	0.684

Table S1E—Proteinuria (spot urine protein)

Variable	Not tested	Tested	p-value
Age (years)	n=1,375; 39.46 ± 14.26	n=141; 48.44 ± 18.17	<0.001
Hemoglobin (g/dL)	n=1,372; 11.78 ± 1.63	n=139; 11.29 ± 1.38	<0.001
Ferritin	n=890; 73.19 ± 116.31	n=116; 87.10 ± 96.25	0.0217
Hypertension	28 (2.0%)	11 (7.8%)	<0.001
CHF	6 (0.4%)	7 (5.0%)	<0.001
Diabetes mellitus	22 (1.6%)	15 (10.6%)	<0.001
Male sex	405 (29.5%)	30 (21.3%)	0.041
Female sex	970 (70.5%)	111 (78.7%)	0.041

Table S1F—Proteinuria (24-hour urine collection)

Variable	Not tested	Tested	p-value
Age (years)	n=1,436; 40.10 ± 14.73	n=80; 43.75 ± 17.32	0.033
Hemoglobin (g/dL)	n=1,433; 11.76 ± 1.63	n=78; 11.28 ± 1.21	0.01
Ferritin	n=944; 75.06 ± 116.34	n=62; 70.67 ± 75.46	0.77
Hypertension	35 (2.4%)	4 (5.0%)	0.159
CHF	11 (0.8%)	2 (2.5%)	0.102
Diabetes mellitus	31 (2.2%)	6 (7.5%)	0.003
Male sex	424 (29.5%)	11 (13.8%)	0.002
Female sex	1,012 (70.5%)	69 (86.3%)	0.002
